# A Possible Link of Genetic Variations in ER/IGF1R Pathway and Risk of Melanoma

**DOI:** 10.3390/ijms21051776

**Published:** 2020-03-05

**Authors:** Tze-An Yuan, Vandy Yourk, Ali Farhat, Katherine L. Guo, Angela Garcia, Frank L. Meyskens, Feng Liu-Smith

**Affiliations:** 1 Program in Public Health, University of California Irvine, Irvine, CA 92697, USA; tzeany@uci.edu (T.-A.Y.); flmeyske@hs.uci.edu (F.L.M.); 2 Department of Neurobiology and Behavior, School of Biological Sciences, University of California Irvine, Irvine, CA 92697, USA; vandyyourk@gmail.com; 3 Department of Biomedical Engineering, The Henry Samueli School of Engineering, University of California Irvine, Irvine, CA 92697, USA; farhatam@uci.edu; 4 Department of Ecology and Evolutionary Biology, University of California Los Angeles, Los Angeles, CA 90024, USA; kathyg9815@g.ucla.edu; 5 Department of Medicine, School of Medicine, University of California Irvine, Irvine, CA 92697, USA; agarcia5@uci.edu; 6 Chao Family Comprehensive Cancer Center, Irvine, CA 92697, USA; 7 Department of Epidemiology, School of Medicine, University of California Irvine, Irvine, CA 92697, USA

**Keywords:** cutaneous melanoma, gender disparities, estrogen receptors, insulin-like growth factor 1, insulin-like growth factor 1 receptor, genetic variants

## Abstract

The mechanism of gender disparity in cutaneous melanoma incidence remains unclear. Steroid hormones including estrogens have long been implicated in the course of melanoma, but the conclusion is controversial. Estrogen receptors (ERs) and insulin-like growth factor 1 receptor (IGF1R) show extensive crosstalk in cancer development, but how the ER/IGF1R network impacts melanoma is currently unclear. Here we studied the melanoma associations of selected SNPs from the ER/IGF1R network. Part of the International Genes, Environment, and Melanoma (GEM) cohort was used as a discovery set, and the Gene Environment Association Studies Initiative (GENEVA) dataset served as a validation set. Based on the associations with other malignant disease conditions, thirteen single nucleotide polymorphism (SNP) variants in ESR1, ESR2, IGF1, and IGF1R were selected for candidate gene association analyses. The rs1520220 in IGF1 and rs2229765 in IGF1R variants were significantly associated with melanoma risk in the GEM dataset after Benjamini-Hochberg multiple comparison correction, although they were not validated in the GENEVA set. The discrepancy may be caused by the multiple melanoma characteristics in the GEM patients. Further analysis of gender disparity was carried out for IGF1 and IGF1R SNPs in the GEM dataset. The GG phenotype in IGF1 rs1520220 (recessive model) presented an increased risk of melanoma (OR = 8.11, 95% CI: 2.20, 52.5, *p* = 0.006) in men but a significant opposite effect in women (OR = 0.15, 95% CI: 0.018, 0.86, *p* = 0.045). The AA genotype in IGF1R rs2229765 (recessive model) showed a significant protective effect in men (OR = 0.24, 95% CI: 0.07, 0.64, *p* = 0.008) and no effect in women. Results from the current study are warranted for further validation.

## 1. Introduction

According to the Surveillance, Epidemiology, and End Results Program (SEER, National Cancer Institute) in their 2019 Annual Report [1], the incidence of cutaneous melanoma (CM) increased in both men and women in the US between 2011 and 2015. CM ranked second in the national trends in rates of new cancer cases in both genders [1]. We and others have shown that there is a distinct age- and sex-dependent incidence pattern of CM [2,3,4,5,6]. Specifically, the incidence rates are higher in older men (> ~ 50 years) but lower in younger men as compared to women of the same ages [5,7]. Younger women (<50 years) showed a faster increase in incidence rates of CM [8].

The gender disparity of CM was reported in the 1970s. A number of hypotheses have been proposed, ranging from biological explanations to sun-protective behaviors. The higher incidence rate in men after the age of 50 was attributed to a lack of awareness in this senior population [9]. Steroid hormones, especially estrogens, have been continuously suggested in CM development in younger women (<50 years) [10]. Pregnant women with a CM diagnosis usually showed poorer prognoses [11]. However, it is still controversial whether oral contraceptives were associated with an increased risk of melanoma or not [12,13,14].

Estrogen initiates its effects mainly through binding to the two major estrogen receptors: ERα (encoded by ESR1 gene) and ERβ (encoded by ESR2 gene) and an additional non-canonical receptor G protein-coupled estrogen receptor (GPER) [15]. The ER genomic pathway refers to the transcriptional activation of ER targeting genes triggered by estrogen/ER binding in the nucleus [16]. In the nongenomic pathway, estrogen binds to membrane-bound ER to initiate signaling transduction such as the MAPK and PI3K pathways in the target cells [16]. Functions of the major ERs vary in different cancer types. ERα variants were associated with breast cancer risk in different racial groups [17,18,19,20]. Loss of ERα was found to be associated with advanced endometrial cancer [21]. In contrast, ERβ overexpression restored the protective role of estrogen, inhibiting cell proliferation in colorectal cancer [22,23]. In CM lesions where melanoma cells had spread to the sentinel lymph nodes, ERα was the predominant receptor [24], while ERβ was predominant in benign melanomas and normal skin [25]. ERα was not found in any benign melanocytic lesions [26].

Moreover, ERs can be activated independently of estrogen when it is coupled to the IGF/IGFR signaling. For instance, ER in the nucleus can be induced by insulin-like growth factor 1 (IGF1) and lead to MAPK signaling activity in the neuroblastoma cells via the genomic pathway [27]. Additionally, estrogen is able to initiate the IGF1 signaling pathway by inducing the expression of IGF1R and its downstream signaling messenger insulin receptor substrate (IRS) [28], which further activates the downstream PI3K signaling pathway [29]. Hence, ER and IGF1R respond to shared ligands (17β-estradiol and IGF1), activate the same downstream signal pathways, and lead to cell proliferation [30]. These molecular events are, however, poorly understood in CM.

The present study used a gene-prioritization approach and aimed to explore the association between single nucleotide polymorphisms (SNPs) in ER/IGF1R-related genes and the risk of melanoma, with a special focus on gender differences. We first genotyped the selected SNPs in cases and controls from the International Genes, Environment, and Melanoma (GEM) Study, which served as our exploratory dataset to prioritize the top SNPs. We later attempted to validate the top SNPs in a second large dataset, the Gene Environment Association Studies Initiative (GENEVA) dataset. Of the thirteen selected SNPs examined, IGF1 rs1520220 and IGF1R rs2229765 SNPs might appear to be significantly associated with melanoma risk in men but not in women.

## 2. Results

### 2.1. Study Participants

Table 1 summarizes the 349 participants from the International Genes, Environment, and Melanoma Study (GEM) [31,32] collected in Southern California and 3114 study subjects from the Gene Environment Association Studies Initiative (dbGaP GENEVA, details are in the Materials and Methods Section 4.2) dataset with available phenotypes [33]. In the GEM dataset, cases and controls were approximately 1:1 matched by age and gender, whereas in the GENEVA dataset, patient numbers were roughly 2:1 to the healthy controls. There were more women cases in younger ages (<50 years) in both datasets, similar to the age distribution of CM in the general population. The family history of melanoma was higher in the controls than in the cases in the GENEVA dataset (63.4% vs. 32.5%, *p* < 0.0001). However, a considerable percentage of patients (27.7% of men and 20.9% of women cases) declared an unknown status of their family history of melanoma. Nearly all of the control subjects submitted a known status of their family history (Table 1). In contrast, there was a significantly higher number of cases having a family history of melanoma as compared to the controls (16.8% vs. 2.3%, *p* < 0.0001) in the GEM dataset.

### 2.2. SNP Selection

Thirteen candidate SNPs were selected based on their associations with various diseases, particularly cancer (Table 2). SNPs rs12662670, rs2234693, rs2046210, rs3734805, and rs827421 in ESR1 were widely studied of their associations with breast cancer risk [8,17,19,20,34,35,36,37,38,39]. ESR2 was also found to be related to various cancer types. For instance, rs1255998 and rs1256061 were linked to lung tumors [40], and rs1256049 was correlated with increased risks of colorectal cancer [41] and prostate cancer [42]. SNPs rs1520220, rs2946834, and rs5742694 in IGF1 had continuously been linked to cancer prognosis [43], as well as coronary artery disease [44]. SNPs rs2229765 and rs8038415 in IGF1R had been linked to several cancer types, including colorectal cancer, breast cancer, papillary thyroid carcinoma, and non-small cell lung cancer [45,46,47,48].

### 2.3. Genotyping and SNP Associations in the GEM Cohort

In order to examine the possible associations between the 13 selected SNPs and risk of melanoma, we used a high throughput PCR-based method to genotype these SNPs in the GEM cohort as an initial exploratory step. The GEM patient samples were originally acquired for the International Genes, Environment, and Melanoma (GEM) study [31,58]. Genotyping calls were machine-validated and then manually examined, as described previously [59]. Four genetic models are considered: genotypic, allelic, recessive, and dominant models. The genotypic model considers the number of minor alleles in a genotype (e.g., aa, Aa, and AA represents the highest, the medium, and the lowest risk, respectively), while the allelic model only considers the presence of one allele (i.e., the counts of A vs. a in the cases and controls) in each group [60]. The dominant model assumes the minor allele (i.e., the presence of one minor allele in the genotype) leads to the disease, while the recessive model requires homozygous minor alleles to be associated with the disease. All models assume the minor allele is associated with the disease due to its lower frequency in the population [61].

The Hardy-Weinberg equilibrium (HWE) test was performed using the GEM control cohort to exclude SNPs significantly deviated from equilibrated genotypes (*p* < 0.05). Three SNPs (rs12662670 and rs2234693 from ESR1 and rs5742694 from IGF1) were thus excluded for further analyses, even though their χ^2^ statistics showed significant association with melanoma risk (Table 3). The screening criterion was set at a *p*-value < a Benjamini–Hochberg critical value [62] to prioritize the SNPs with significant associations with melanoma. Benjamini–Hochberg multiple comparison correction was used at this step at a false discovery rate of 0.25 by default [63]. Association of alleles or genotypes with melanoma risk were tested by the χ^2^ test of independence based on genotypic, allelic, recessive, and dominant genetic models (Table 3). IGF1R rs2229765 (*p* = 0.004) and IGF1 rs1520220 (*p* = 0.005) showed significant genotypic and recessive differences between melanoma cases and healthy controls (Table 3).

### 2.4. An Attempt to Validate the Top 2 SNPs in the GENEVA Dataset

In order to validate our findings from the GEM dataset, we extracted genotyping data from the GENEVA dataset (Table 4). Quality control (QC) for the GENEVA dataset was performed in two stages, including per-individual QC and per-SNP QC. The initial per-individual QC report was attached to the GENEVA dataset (details are in the Materials and Methods Section 4.4). Of the 3114 study subjects (Table 1), genotyping data were available for 3110 individuals. Per-individual QC reported 68 duplicated individuals and an additional 17 individuals with familial relationships. The rest of the 3025 individuals were further examined for genotyping intensity and chromosomal anomalies (i.e., genotyping errors). Three thousand and three (1965 cases and 1038 controls) study subjects passed this level of QC and were used for further analyses. The genotyping call rate reached 99.9%.

All 13 SNPs were analyzed in the GENEVA dataset in an attempt to gain a complete understanding of the ESR/IGF1 pathway. As shown in Table 4, the melanoma associations of IGF1R rs2229765 and IGF1 rs1520220 were not replicable in the GENEVA dataset. On the other hand, SNPs rs2234693 (*p* = 0.035 < 0.038 critical value) and rs827421 (*p* = 0.018 < 0.019 critical value) in ESR1 had only borderline Benjamini–Hochberg corrected significant genotypic differences between melanoma cases and healthy controls in the GENEVA dataset (Table 4). The minor alleles also fitted in a dominant genetic model (*p* = 0.010 and 0.005, respectively). However, these two SNPs were not observed in the GEM discovery set. It was noted that rs2234693/ESR1 did not present a significant Benjamini–Hochberg corrected association with melanoma risk in the GEM set might be a result of HWE deviation.

Since estrogen is able to initiate the IGF1 signaling pathway by inducing the expression of IGF1R and its downstream signaling that leads to cell proliferation [30], the IGF1 and IGF1R SNPs might still provide crucial information on the ER/IGF1R network in melanoma. Moreover, as described in one of our previous publications [59], all patients in the GEM set had multiple melanomas which might provide unique genetic information on melanoma risk. The IGF1 rs1520220 and IGF1R rs2229765 SNPs were thus further analyzed in the logistic regression models to determine the odds ratio (OR) of melanoma in individuals carrying minor alleles/genotypes in comparison with the reference alleles/genotypes (Table 5).

In the unadjusted crude regression analyses (model A), only the recessive genetic models showed significant associations with melanoma: the OR of GG v.s. CC + CG reference genotypes in rs1520220/IGF1 was 2.97 (95% CI: 1.35, 7.23, *p* = 0.010, model likelihood *p* = 0.006); the OR of AA vs. GG + GA reference genotypes in rs2229765/IGF1R was 0.29 (95% CI: 0.12, 0.62, *p* = 0.003, model likelihood *p* = 0.001) (Table 5). Therefore, due to the complexity of the regression analyses based on different genetic models (i.e., additive/genotypic, recessive, and dominant models), hereafter we only presented the recessive genetic model results of IGF1 and IGF1R as indicated by the unadjusted crude results in Table 5 and the *χ*^2^ test results in Table 3.

Additional regression analyses were adjusted by gender (model B), family history of melanoma (model C), or both (model D). The odds ratio estimation from the gender-adjusted model B: OR 2.86 (95% CI: 1.29, 6.98, *p* = 0.014) was slightly reduced for rs1520220/IGF1 as compared to model A. On the contrary, the odds ratio estimation remained the same for rs2229765/IGF1R in model B: OR 0.29 (95% CI: 0.12, 0.63, *p* = 0.003). It was noted that the coefficients of gender did not show significance for both SNPs. However, the overall model significance remained significant (*p* = 0.009 for rs1520220/IGF1 and *p* = 0.001 for rs2229765/IGF1R).

When the family history of melanoma was taken into consideration, the odds ratio estimations were further reduced for both SNPs. In the family history of melanoma-adjusted recessive models, the OR for rs1520220/IGF1 was 2.79 (95% CI: 1.24, 6.92, *p* = 0.018) in model C and approximately the same in model D. The OR for rs2229765/IGF1R was 0.25 (95% CI: 0.10, 0.57, *p* = 0.002) in model C and remained the same in model D. This might be because of the coefficients of family history of melanoma were highly significant in both models C and D for both SNPs and might confound the SNP results. However, this confounding effect was not considered severe because the OR differences were less than 10% [64] for both SNPs between models (rs1520220/IGF1 crude OR: 2.97 → model C OR: 2.79; rs2229765/IGF1R crude OR: 0.29 → model C OR: 0.25).

### 2.5. Gender Disparity of the Association

Lastly, the GEM cohort was stratified by gender and logistic regression analyses were performed to measure the associations of rs1520220/IGF1 and rs2229765/IGF1R and melanoma risk in men and women, respectively. Surprisingly, the melanoma associations were only significant in men for both SNPs.

For rs1520220/IGF1 in the recessive model, men with GG genotype showed extremely high risk of melanoma (OR 8.11, 95% CI: 2.20, 52.50, *p* = 0.006) while women with GG genotype showed a significant lower risk (OR 0.15, 95% CI: 0.018, 0.86, *p* = 0.045). For rs2229765/IGF1R, men with AA genotype presented a significant lower risk (OR 0.24, 95% CI: 0.07, 0.64, *p* = 0.008), while AA in women did not show a significant association (Table 6).

### 2.6. eQTL of the Three SNPs in Skin Tissue

**Figure 1:**
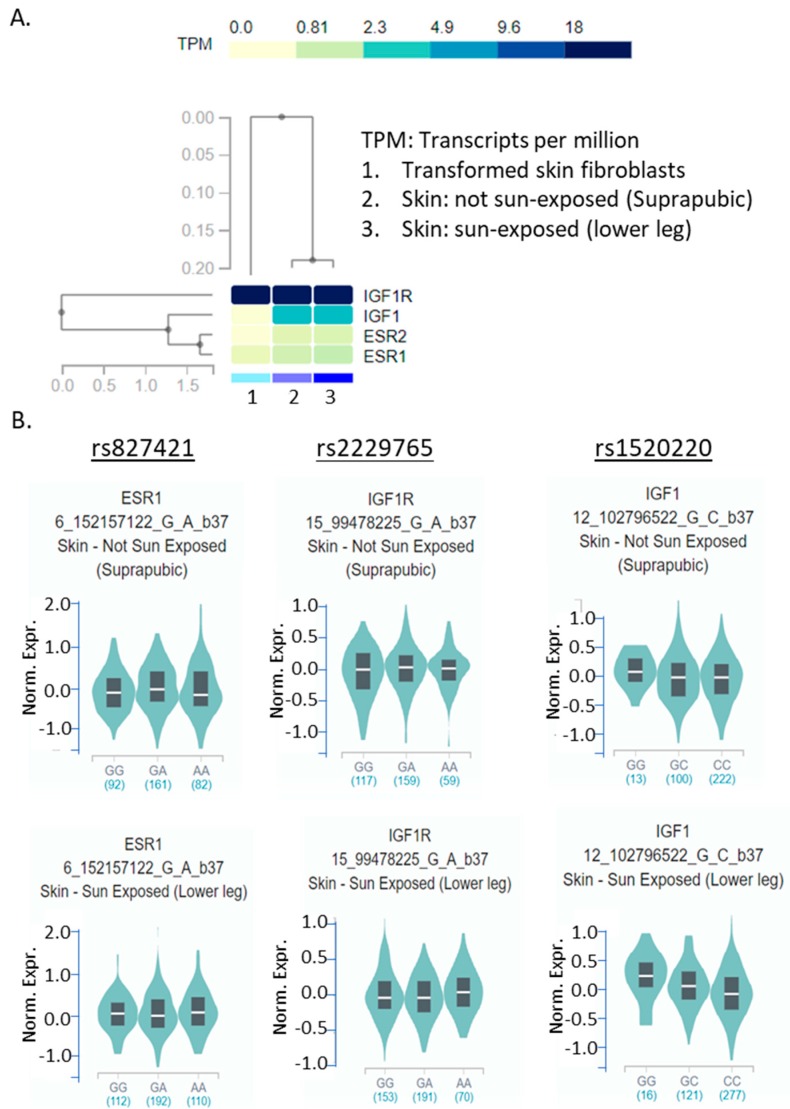
eQTL of the 4 genes and 3 SNPs in skin tissues. (**A**). Multiple gene queries in skin tissues. (**B**). Violin plot of each SNP (underlined rs numbers) in sun-exposed and not-exposed skin. Sun-exposed skin was derived from lower legs, while not-exposed skin was obtained from suprapubic areas. Data analysis was performed using GTEx Portal and included tissue-specific mRNA expression information provided by the website (https://gtexportal.org/home/).

Expression of ESR1 and ESR2 in melanoma progression has been controversial. In general, melanoma cells may express low level of ERα but may retain expression of ERβ [24,25,26]. IGF1 serum levels were significantly higher in melanoma patients and correlated with Breslow depth, identified as a prognosis factor [65], but a recent large study did not find a correlation of IGF1 serum level with melanoma risk [66]. However, IGF1 requires receptors such as IGF1R to trigger a signal cascade. IGF1R is known to play an important role in cancer development and progression [30]. We used the Broad Institute GTEx Portal (https://gtexportal.org/home/) to map the expression levels of the four genes in skin tissues (Figure 1A). As expected, IGF1R showed high expression in skin tissues as well as in transformed skin fibroblasts. Expressions of ESR1 and ESR2 were low in skin tissues but still higher than that in transformed skin fibroblasts (Figure 1A). It is worth to point out that as melanocytes only represent a very small cell population in skin tissues (one melanocyte is surrounded by ~30–40 keratinocytes) [67], the mRNA expression in skin tissues may not fully reflect gene expression in melanocytes. Next, we also determined the eQTL (expression quantitative trait loci) of the three important SNPs in skin tissues (Figure 1B). The mRNA expression levels of ESR1 or IGF1R were not associated with genotypes in rs827421 or rs2229765 (*p* = 0.40 and 0.55 in sun-exposed skin, respectively). Interestingly, the mRNA level of IGF1 showed a significant association with genotypes in rs1520220 in the sun-exposed skin (*p* = 0.00023) but not in the not-exposed skin (*p* = 0.27). The homozygous minor alleles GG showed higher expression than the other two genotypes (Figure 1B). This may raise an interesting question of whether sun-exposure modifies IGF1 expression based on an individual’s genotype.

## 3. Discussion

In an attempt to understand the genetic predisposition to the gender-biased risk of cutaneous melanoma, a gene prioritization approach and case-control study design were used to measure melanoma associations with a group of 13 SNPs from the ER/IGF1R pathway. Genotyping of SNPs was carried out in the GEM cohort originated from Southern California. The most significant two SNPs (IGF1R SNP rs2229765 and IGF1 SNP rs1520220) were further examined in the large GENEVA cohort originally recruited by MD Anderson Cancer Center, Huston, Texas. After Benjamini–Hochberg multiple testing correction, IGF1R SNP rs2229765 and IGF1 SNP rs1520220 failed to be validated in the GENEVA cohort. On the other hand, ESR1 SNPs rs2234693 and rs827421 only slightly presented Benjamini–Hochberg significances of their genotypic and dominant genetic model differences between melanoma cases and controls in the GENEVA cohort. Nevertheless, as the GEM cohort included multiple melanoma patients [59] that might provide unique genetic information, the IGF1R SNP rs2229765 and IGF1 SNP rs1520220 were further analyzed in regression models. Multiple logistic regression models later revealed that the G allele in IGF1 rs1520220 carriers exhibited a higher risk of melanoma as compared to the reference C allele in a recessive genetic model, while the A allele in IGF1R rs2229765 showed a protective effect comparing to the reference G allele in the recessive genetic model. These effects were, however, only shown significances in the male cohort but not in females.

IGF1 SNP rs1520220 first appeared in the literature in 2005. The C allele of rs1520220 was found significantly associated with increased serum IGF1 levels and an increased risk of breast cancer in women [68]. Later, the rs1520220 C allele was widely discussed in increasing different types of cancer risks and IGF1 serum levels, such as prostate cancer [69,70], ovarian cancer [71], stomach cancer [72], and more. Of these studies, none have been addressed in melanoma, and hence, the actual causal mechanism of IGF1 rs1520220 in melanoma remains unclear. Nevertheless, IGF1 is one of the essential growth factors known for its direct carcinogenesis effect by activating the PI3K/Akt mitogenesis, cell cycle protection, and anti-apoptosis pathway through binding to IGF1R [73]. Indirectly, IGF1 works with sex hormones to intensify these cancerous activities, including cell proliferation, transformation, and metastasis [73]. Our current results also showed an increased risk of melanoma by IGF1 rs1520220 C alleles. This effect was further magnified in the male cohort, but an opposite association was found in females. Perhaps the indirect effect of IGF1 is playing a role in gender disparities in melanoma, which awaits further laboratory studies to reveal any gender-specific crosstalk between IGF1 rs1520220 and sex hormones in melanoma.

In contrast, the A alleles in IGF1R SNP rs2229765 presented a protective effect on melanoma in the current study. The current understanding of this SNP in the literature is controversial. For instance, the A allele was found associated with advanced colorectal cancer [46] and an increased risk of breast cancer [74]. On the contrary, the A alleles showed no association with non-small cell lung cancer survival [75], IGF1R expression, or breast cancer survival [76]. Interestingly, the A allele was discovered to be protective in papillary thyroid carcinoma [45]. rs2229765 SNP G > A is known to be a “silent” mutation, which means the nucleotide acid change from guanine to adenosine at this locus does not change the encoded protein and is thus not likely to be a functional alteration [76]. From experimental animal studies, IGF1R showed a pivotal role in the development of ovaries and fertility in female mice [77]. This gender-specific activity was also seen in breast cancer cells where estradiol (a female-sex hormone) interacts with IGF1R to adhere to extracellular matrices as a marker of cancer progression [78]. This perhaps could be a reason why our result in females also showed an increased risk of melanoma by IGF1R rs2229765, although the result was nonsignificant, and its association with breast cancer is still inconclusive in the literature. On the other hand, IGF1R usually co-expressed with androgen receptor in response to dihydrotestosterone (a male-sex hormone)-dependent prostate cancer cell proliferation [79]. While this silent mutation of rs2229765 polymorphism favored longevity in male carriers of the homozygous A alleles [80], the protective effect of IGF1R rs2229765 in the current study in men remains an interesting focal point in future melanoma gender disparity studies.

The IGF1 SNP rs1520220 and IGF1R SNP rs2229765 were identified in the GEM cohort (Table 3) but not in the GENEVA cohort (Table 4). Similarly, ESR1 SNPs rs2234693 and rs827421 were not observed significantly in the GEM cohort. A possible explanation for this nonreplication on genotype frequencies is perhaps because the patient characteristics in these two datasets are very different. In the GENEVA cohort, only newly diagnosed malignant melanoma cases were eligible and recruited. Of these patients, only 2.8% developed more than one primary tumor. However, the GEM cohort included a large portion of patients with multiple melanoma tumors (22.0%) [31,58]. The ESR1 SNPs are still likely to play a role in melanoma development. Indeed, we have found a universal expression of an ESR1 isoform ERα36 in melanocytes and melanoma cells, which may further explain the importance of ESR1 SNPs rs2234693 and rs827421. Therefore, these two SNPs also warrant further investigations.

A major limitation of this current study was the high missing rate of family history of melanoma status in the GENEVA patients as shown in Table 1. Approximately 27.7% of men and 20.9% of women showed an unknown status of their family history, which may influence the results from the adjusted models and decrease the precision of OR estimation. On the other hand, almost all controls reported a family history of melanoma (Table 1), which may also add biases to the results. A minor limitation was the relatively smaller sample size in the validation cohort of the GEM study, which may not provide sufficient power for the targeted SNPs, although the primary reason for nonreplication may likely be the patient composition differences between these two cohorts, as described in the paragraph above. One additional reason for why we could not replicate the samples in these two datasets may lie in the SNP genotyping step. We genotyped the GEM samples in our lab [59], using two alleles only; while the SNPs may have up to four alleles in one location (i.e., T, C, G, and A). The GENEVA samples were potentially able to call all potential SNPs, and therefore, this might be another reason that we could not replicate the findings. Nevertheless, as demonstrated in our series of publications [2,3,4,7,81], the gender difference in melanoma incidence is significant but not yet fully explained. Meanwhile, studies on hormone impact on melanoma are still ongoing in our lab and other research groups. Taken together, this current study will certainly help us form hypotheses that aid in future research.

In summary, our data might suggest that the G allele in IGF1 rs1520220 is likely to be associated with melanoma risk, while the A allele in IGF1R rs2229765 may have a protective effect, especially in men, in recessive genetic models. The ESR1 SNPs rs2234693 and rs827421 may play a role in melanoma patients, but further analysis is needed. These findings may provide some understanding of gender-specific melanoma risks. However, the molecular mechanisms will require further investigation in order to completely dissect the role of the ER/IGF1R pathway in cutaneous melanoma development.

## 4. Materials and Methods

### 4.1. Ethics Statement

We obtained approval from the Institutional Review Board of the University of California Irvine Office of Research (protocol number 2011-8238, approved 27 June 2011) for the use of the samples from the International Genes, Environment, and Melanoma (GEM) study. An Institutional Review Board was not required for the use of the data provided by the U.T. M.D. Anderson Cancer Center, in which the data were part of the Gene Environment Association Studies Initiative (GENEVA, http://www.genevastudy.org) funded by the trans-NIH Genes, Environment, and Health Initiative (GEI). However, authorized access to the dataset was required by dbGaP.

### 4.2. Study Population

The GEM study was described before [31] and served as the discovery set. The dbGaP GENEVA dataset contains samples of 2054 European ancestry melanoma cases enrolled between 1994 and 2006 at the U.T. M.D. Anderson Cancer Center (Table 1). Friends or spouses of these enrolled cases (1060 individuals) were recruited as controls. The exclusion criteria included a history of prior cancers (other than skin cancer). Only the Caucasian subjects from both datasets were included for analysis.

### 4.3. Genotyping and Quality Control

DNA was extracted from buccal cell samples using the Qiagen DNA extraction kit (Germantown, MD, USA). PCR-based genotyping was performed using 384-well plates and the Applied Biosystems ViiA 7 system. Gene-specific primers were custom-designed by Qiagen. Genotyping calls were initially performed by the QuantStudio™ 6 Flex Real-Time PCR System. Per-SNP quality control (QC) for the GEM samples was performed as previously described [59]. Quality control for the GENEVA dataset was completed in two stages, including per-individual QC and per-SNP QC, as described in the main text. Plink (v1.90b6.2) was used to examine missing data in sex and genotyping (< 3%), as well as familial relationships. Overall, the genotyping call rate reached 99.887%. In terms of per-SNP QC, we performed SNP missingness (< 5%), Hardy-Weinberg Equilibrium (> 1 × 10^−4^, GENEVA recommendation), and minor allele frequency checks (MAF ≥ 5%). SNP number dropped from 1,012,904 to 739,936, in which rs827421 was included. Imputed SNPs were provided in the GENEVA dataset downloaded from dbGaP, and thus, imputed SNPs were included for analyses, such as rs1520220 and rs2229765.

### 4.4. Statistics

Computational analyses were performed by using Plink (v1.90beta6.2, 12 June 2018, http://zzz.bwh.harvard.edu/plink/), Plink2.00alpha (29 June 2018, specifically for imputation analysis, https://www.cog-genomics.org/plink/2.0/) in the high-performance computing cluster (University of California Irvine, https://hpc.oit.uci.edu/) and RStudio (v1.1.453). *χ*^2^ test of independence was performed to examine the associations between SNP candidates and melanoma case-control status. Statistical significance was adjusted by the Benjamini–Hochberg procedure to correct multiple comparisons, using a false discovery rate of 25% [62,63]. Simple logistic regression models showing the crude odds ratios between the binary response variable (melanoma case-control status), and primary study variables of interest (filtered SNPs) were conducted separately based on additive, recessive, and dominant allele models. Dummy variables of the SNPs in the models were created by default, making the genotype with homozygous major alleles as the reference. The family history of melanoma—which was one of the confounders necessarily to be adjusted as previously described by us [59]—was controlled in the multiple logistic regression models. The gender effect was evaluated by confounding adjustment and effect stratification in the logistic regression models. The lambda value [82] of population stratification was computed to be 1.01 (as close as to be 1.00) in the GENEVA dataset, and thus, population stratification was not considered necessary to be controlled in the regression models. Indeed, >99% of the patients in the GENEVA dataset were European ancestry whites [83], and we did not include any racial minority subjects in the present study.

## Figures and Tables

**Table 1:** Characteristics of the study participants.

Study Participants	GEM (*N* = 349)								GENEVA (*N* = 3114)							
	Cases (*N* = 177)				Controls (*N* = 172)				Cases (*N* = 2054)				Controls (*N* = 1060)			
	Men		Women		Men		Women		Men		Women		Men		Women	
	*N*	%	*N*	%	*N*	%	*N*	%	*N*	%	*N*	%	*N*	%	*N*	%
Age (years)																
0–29	0	0	2	1.1	0	0.0	0	0	55	2.7	100	4.9	16	1.5	46	4.3
30–49	13	13.8	28	15.8	20	11.6	37	21.5	373	18.2	352	17.1	198	18.7	184	17.4
50–69	43	24.3	37	20.9	59	34.3	29	16.9	606	29.5	336	16.4	366	34.5	186	17.5
70+	33	18.6	12	6.8	19	11.0	8	4.7	153	7.4	79	3.8	48	4.5	16	1.5
Unknown	5	2.8	4	2.3	0	0.0	0	0	0	0	0	0	0	0	0	0
Family history of melanoma																
Yes	10	5.6	18	10.2	1	0.6	3	1.7	388	18.9	280	13.6	399	37.6	273	25.8
No	78	44.1	60	33.9	97	56.4	71	41.3	231	11.2	157	7.6	227	21.4	159	15.0
Unknown	6	3.4	5	2.8	0	0.0	0	0.0	568	27.7	430	20.9	2	0.2	0	0.0
Total	94	53.1	83	46.9	98	57.0	74	43.0	1187	57.8	867	42.2	628	59.2	432	40.8

**Table 2:** Selection of the 13 candidate SNPs.

Gene	SNP	Location	dbSNP ID	Minor Allele Disease Associations	References
*ESR1*	T > G	Intron	rs12662670	Common breast cancer locus	[19]
	−397T > C	Promoter	rs2234693	breast cancer susceptibility, prostate cancer risk	[49,50]
	G > A	Promoter	rs2046210	Breast cancer risk	[36,37]
	A > C	Promoter	rs3734805	Breast cancer risk	[36]
	A > G	Intron	rs827421	Breast cancer risk	[51]
*ESR2*	C > G	3’UTR	rs1255998	Endometrial cancer	[52]
	C > T	Exon	rs1256049	Risks of breast cancer and colorectal cancer	[41,53]
	G > A	Intron	rs1256061	Risks in lung tumors and ovarian cancer	[40,54]
*IGF1*	C > G	Intron	rs1520220	Obesity, poor breast cancer survival, pancreatic cancer risk	[43,55,56]
	A > G	Intron	rs2946834	Poor outcome in patients with breast cancer	[43]
	C > A	Intron	rs5742694	Colorectal cancer risk, poor breast cancer survival	[43,57]
*IGF1R*	G > A	Exon	rs2229765	Colorectal cancer risk, papillary thyroid carcinoma risk	[45,46]
	T > C	Intron	rs8038415	Risks in non-small cell lung cancer, breast cancer	[47,48]

**Table 3:** Association of the 13 SNP candidates with melanoma in the Genes, Environment, and Melanoma (GEM) dataset.

SNP	Gene	Genotyping Rate ^a^		Minor Allele Frequency (MAF)		Association (*p*-Value | Benjamini–Hochberg Critical Value) ^b^				HWE ^c^ (*p*-Value)	dbSNP MAF ^d^
		Cases (*n* = 170)	Controls (*n* = 152)	Cases (*n* = 170)	Controls (*n* = 152)	Genotypic	Allelic	Recessive	Dominant		
rs12662670	*ESR1*	97.1%	82.2%	6.7%	9.6%	0.083 | 0.077	0.255 | 0.115	0.034 | 0.077	0.638 | 0.173	0.014	10.7%
rs2046210	*ESR1*	94.7%	88.2%	30.4%	36.6%	0.247 | 0.115	0.137 | 0.058	0.650 | 0.212	0.120 | 0.058	0.458	41.2%
rs2234693	*ESR1*	98.2%	96.1%	38.3%	36.0%	0.254 | 0.135	0.598 | 0.212	0.588 | 0.173	0.238 | 0.077	0.007	44.6%
rs3734805	*ESR1*	98.2%	75.7%	6.3%	7.8%	0.575 | 0.212	0.590 | 0.192	N/A	0.575 | 0.154	1.000	10.5%
rs827421	*ESR1*	98.8%	86.8%	51.2%	59.1%	0.141 | 0.096	0.059| 0.038	0.214| 0.096	0.097 | 0.038	0.857	47.8%
rs1255998	*ESR2*	98.8%	96.1%	8.6%	11.3%	0.349 | 0.154	0.325 | 0.135	0.465 | 0.154	0.370 | 0.115	0.696	36.9%
rs1256049	*ESR2*	97.6%	98.0%	7.8%	7.7%	1.000 | 0.25	1.000 | 0.25	N/A	1.000 | 0.25	1.000	13.0%
rs1256061	*ESR2*	98.2%	88.8%	49.1%	47.4%	0.384 | 0.173	0.740 | 0.231	0.335 | 0.115	0.786 | 0.212	0.731	40.0%
**rs1520220**	***IGF1***	97.1%	88.8%	28.2%	24.8%	**0.005** | 0.058	0.404 | 0.154	**0.013** | 0.038	0.672 | 0.192	1.000	32.0%
rs2946834	*IGF1*	97.6%	74.3%	35.2%	40.7%	0.445 | 0.192	0.222 | 0.077	0.370 | 0.135	0.362| 0.096	0.435	40.0%
rs5742694	*IGF1*	98.8%	93.4%	50.6%	41.2%	< 0.001 | 0.019	0.024| 0.019	0.025 | 0.058	< 0.001 | 0.019	< 0.001	21.6%
**rs2229765**	***IGF1R***	97.6%	96.1%	35.8%	40.8%	**0.004** | 0.038	0.239 | 0.096	**0.003** | 0.019	0.918 | 0.231	1.000	33.6%
rs8038415	*IGF1R*	97.1%	98.1%	51.5%	54.3%	0.654 | 0.231	0.411 | 0.173	0.648 | 0.192	0.458 | 0.135	1.000	42.5%

**^a^** Percentage of participants with successful SNP genotyping. **^b^** Chi-square test of independence between SNP models and melanoma case-control status. *p*-value < Benjamini–Hochberg critical value for multiple comparison correction counts as statistically significant. The false discovery rate for Benjamini–Hochberg procedure is at 0.25 by default. SNPs that are showing statistical significance are bolded. **^c^** Exact test for Hardy-Weinberg equilibrium (HWE) using the control samples only. *p*-value < 0.05 counts as evidence of unequilibrated genotypes. ^d^ Reference minor allele frequencies documented in the NCBI dbSNP database.

**Table 4:** Association of the 13 SNP candidates with melanoma in the Gene Environment Association Studies Initiative (GENEVA) dataset.

SNP ^a^	Gene	Minor Allele Frequency (MAF)		Association (*p*-Value | Benjamini–Hochberg Critical Value) ^b^				HWE ^c^ (*p*-Value)	dbSNP MAF ^d^
		Cases (*n* = 1965)	Controls (*n* = 1038)	Genotypic	Allelic	Recessive	Dominant		
rs12662670 ^e^	*ESR1*	14.1%	14.3%	0.995 | 0.231	0.446 | 0.212	0.961 | 0.231	0.069 | 0.077	0.997	10.7%
rs2046210 ^e^	*ESR1*	54.2%	51.1%	0.640 | 0.135	0.248 | 0.135	0.936 | 0.212	0.155 | 0.154	0.984	41.2%
**rs2234693**	***ESR1***	47.2%	44.4%	**0.035** | 0.038	0.047 | 0.038	0.580 | 0.077	**0.010** | 0.038	0.059	44.6%
rs3734805	*ESR1*	8.0%	6.9%	0.181 | 0.096	0.129 | 0.115	0.660 | 0.115	0.090 | 0.115	0.624	10.5%
**rs827421**	***ESR1***	50.7%	47.6%	**0.018** | 0.019	0.027 | 0.019	0.440 | 0.058	**0.005** | 0.019	0.192	47.8%
rs1255998	*ESR2*	8.9%	10.5%	0.093 | 0.058	0.048 | 0.058	0.110 | 0.019	0.079 | 0.096	0.869	36.9%
rs1256049	*ESR2*	2.8%	3.5%	NA	0.112 | 0.096	NA	0.104 | 0.135	0.634	13.0%
rs1256061 ^e^	*ESR2*	60.7%	62.6%	0.971 | 0.212	0.404 | 0.192	0.678 | 0.154	0.581 | 0.231	0.979	40.0%
rs1520220 ^e^	*IGF1*	63.7%	62.6%	0.752 | 0.173	0.281 | 0.154	0.642 | 0.096	0.257 | 0.173	0.985	32.0%
rs2946834	*IGF1*	31.2%	33.2%	0.158 | 0.077	0.102 | 0.077	0.672 | 0.135	0.055 | 0.058	0.043	40.0%
rs5742694 ^e^	*IGF1*	65.0%	65.5%	0.941 | 0.192	0.283 | 0.173	0.740 | 0.173	0.263 | 0.192	1.000	21.6%
rs2229765 ^e^	*IGF1R*	59.7%	57.2%	0.315 | 0.115	0.950 | 0.25	0.403 | 0.038	0.417 | 0.212	1.000	33.6%
rs8038415 ^e^	*IGF1R*	61.3%	60.0%	0.646 | 0.154	0.815 | 0.231	0.806 | 0.192	0.609 | 0.25	1.000	42.5%

^a^ Ordered by genes according to smallest to largest rs numbering. ^b^ Chi-square test of independence between SNP models and melanoma case-control status. *p*-value < Benjamini–Hochberg critical value for multiple comparison correction counts as statistically significant. The false discovery rate for Benjamini–Hochberg procedure is at 0.25. SNPs that are showing statistical significance are bolded. ^c^ Exact test for Hardy-Weinberg equilibrium (HWE) on the controls. *p*-value < 1 × 10^−4^ counts as evidence of unbalanced genotypes (GENEVA recommendation). ^d^ Reference minor allele frequencies documented in the NCBI dbSNP database. ^e^ Imputed.

**Table 5:** Associations of IGF1 rs1520220 and IGF1R rs2229765 SNPs with melanoma risk in the GEM dataset.

Logistic Regression Models				Crude (Model A)		Model B ^b^		Model C ^c^		Model D ^d^	
SNPs/Genetic Models	Genotypes	Cases *n* (%)	Controls *n* (%)	OR (95% CI)	*p*-Value ^a^	OR (95% CI)	*p*-Value	OR (95% CI)	*p*-Value	OR (95% CI)	*p*-Value
rs1520220/IGF1 Recessive	CC + CG	139 (81.8%)	127 (83.6%)	Reference	--	Reference	--	Reference	--	Reference	--
	GG	26 (15.3%)	8 (5.3%)	2.97 (1.35, 7.23)	0.010	2.86 (1.29, 6.98)	0.014	2.79 (1.24, 6.92)	0.018	2.80 (1.24, 6.93)	0.018
	Sex	--	--	--	--	1.08 (0.68, 1.72)	0.743	--	--	0.95 (0.58, 1.53)	0.819
	Family history	--	--	--	--	--	--	6.60 (2.48, 22.86)	0.0006	6.68 (2.50, 23.26)	0.006
	Model	--	--	--	0.006	--	0.009	--	0.0117	--	0.0117
rs2229765/IGF1R Recessive	GG + GA	157 (92.4%)	122 (80.3%)	Reference	--	Reference	--	Reference	--	Reference	--
	AA	9 (5.3%)	24 (15.8%)	0.29 (0.12, 0.62)	0.003	0.29 (0.12, 0.63)	0.003	0.25 (0.10, 0.57)	0.002	0.25 (0.10, 0.56)	0.002
	Sex	--	--	--	--	0.98 (0.62, 1.56)	0.948	--	--	0.84 (0.52, 1.36)	0.484
	Family history	--	--	--	--	--	--	8.38 (3.08, 29.79)	0.00017	8.77 (3.19, 31.44)	0.00014
	Model	--	--	--	0.001	--	0.001	--	0.002	--	0.002

^a^*P*-value of the coefficient from the regression model. The overall model significance was derived from the likelihood ratio test (*χ*^2^ statistic). ^b^ Model B, adjusted for gender. ^c^ Model C, adjusted for family history of melanoma. ^d^ Model D, adjusted for gender and family history of melanoma.

**Table 6:** Associations of IGF1 rs1520220 and IGF1R rs2229765 with melanoma risk in two gender strata in the GEM dataset.

Model		Male OR (95% CI)	*p*-Value ^a^	Female OR (95% CI)	*p*-Value
rs1520220/IGF1 Recessive	CC + CG	Reference	--	Reference	--
	GG	8.11 (2.20, 52.5)	0.006	0.15 (0.018, 0.86)	0.045
rs2229765/IGF1R Recessive	GG + GA	Reference	--	Reference	--
	AA	0.24 (0.07, 0.64)	0.008	1.70 (0.32, 8.86)	0.526

^a^*p*-value of the coefficient from the regression model.

## Abbreviations

CM	Cutaneous melanoma
SNP	Single nucleotide polymorphism
OR	Odds ratio
ER	Estrogen receptor
IGF1	Insulin-like growth factor 1
GENEVA	The Gene Environment Association Studies Initiative
GEM	The International Genes, Environment, and Melanoma Study
